# Diffuse traumatic brain injury in mice is associated with a transient mismatch of cerebral blood flow and energy metabolism

**DOI:** 10.1177/0271678X251364136

**Published:** 2025-08-13

**Authors:** Sertan Arkan, Michael Gottschalk, Saema Ansar, Jesper Peter Bömers, Johannes Ehinger, Eskil Elmér, Imen Chamkha, Michael Karlsson, Niklas Marklund

**Affiliations:** 1 Department of Clinical Sciences Lund, Neurosurgery, Lund University, Lund, Sweden; 2 Skåne University Hospital, Lund, Sweden; 3 Lund University Bioimaging Centre, 5193 Lund University, Lund, Sweden; 4 Department of Neurosurgery, Rigshospitalet, Copenhagen, Denmark; 5 Mitochondrial Medicine, Department of Clinical Sciences Lund, Lund University, Lund, Sweden; 6 Otorhinolaryngology, Head and Neck Surgery, Department of Clinical Sciences Lund, Lund University, Skåne University Hospital, Lund, Sweden

**Keywords:** Traumatic brain injury (TBI), cerebral blood flow, energy metabolism, mitochondria, central fluid percussion injury, axonal injury

## Abstract

Axonal injuries commonly contribute to poor functional outcomes following traumatic brain injury (TBI). To assess cerebral blood flow (CBF) and energy metabolic disturbances in a TBI model of widespread axonal injury, we exposed 105 adult mice to the central (midline) fluid percussion injury (cFPI) diffuse TBI model, or sham injury, and used 9.4 T magnetic resonance (MR) arterial spin labeling (ASL), cortical and hippocampal mitochondrial respiration, and hippocampal MR spectroscopy at 1- and 7-days post-injury (dpi). Widespread, bilateral CBF reductions were observed at day 1 dpi, changes that were normalized by 7 dpi. However, cortical and hippocampal mitochondrial respiration and reactive oxygen species (ROS) production was not significantly altered at 1 and 7 dpi. Moreover, hippocampal volumes, evaluated by MRI, were not altered by cFPI, and by immunohistochemistry only a few apoptotic hippocampal cells were observed. By MRS, evidence of delayed (7 dpi) membrane disruption (phosphocholine and glycerophosphocholine) and glutamate/glutamine increase were observed. While widespread traumatic axonal pathology associated with functional impairments is observed in this TBI model, early CBF alterations were transient and did not translate into significant energy metabolic disturbances. Instead, the delayed hippocampal metabolite changes observed by MRS may contribute to the functional impairment observed in this diffuse TBI model.

## Introduction

Traumatic brain injury (TBI) is a major health concern worldwide. In Europe alone, TBI is a leading cause of mortality and morbidity, leading to 57.000 TBI-related annual deaths, and 1.5 million TBI-related hospital admissions.^1,2^ Even in mild TBI, patients often experience persistent symptoms such as headache, fatigue, and cognitive impairments.^3^ Moreover, TBI is a markedly heterogeneous disease, characterized by a wide range of pathophysiological differences among TBI subtypes that include both focal TBI (epi- and subdural hematomas, and traumatic intracerebral hematomas/contusions) and diffuse injuries (diffuse edema, traumatic axonal injury).^4,5^ Axonal injury is observed also in focal TBI subtypes, and in milder forms of TBI, and is a key contributor to persistent neurological deficits.^6,7^

TBI typically results in deformation of brain tissue and an immediate primary injury, which is followed by secondary injury processes that include cerebrovascular dysfunction, neuroinflammation, and energy metabolic alterations.^8^ These secondary injury factors may persist for years post-injury, markedly exacerbate the initial injury and result in progressive white matter atrophy^9^ and significantly increasing risk of neurodegenerative disorders.^2,10,11^ In diffuse TBI, immediate axonal transection is rare and observed mainly in the most severe TBI cases.^12^ More often, secondary injury processes contribute to delayed axonal disconnection.^13^ The central (midline) fluid percussion injury (cFPI) is an well-established diffuse TBI model that replicates wide-spread axonal pathology.^14,15^ Since rodent models are unsuitable to mimic severe TBI with prolonged unconsciousness, the cFPI model is commonly used to simulate a mild-moderate TBI.^16,17^ However, secondary injury mechanisms have insufficiently been explored in this TBI model.

Changes in cerebral blood flow (CBF), and the association between CBF and energy metabolic disturbances, have been evaluated using a variety of techniques in different rodent TBI models including the lateral fluid percussion and focal cortical contusion models.^18–21^ In pre-clinical TBI studies using focal or diffuse TBI models, vascular alterations, blood-brain barrier (BBB) disruption,^22^ pericyte loss,^23^ and formation of edema^24^ have been reported. To sustain the physiological functions of the brain by delivering oxygen and nutrients, and removing toxic waste, CBF needs to be tightly regulated and titrated to the energy demands. Vascular alterations induced by TBI may lead to ischemia, energy depletion, and an energy metabolic crisis but have rarely been evaluated in diffuse TBI models.

Although mitochondrial disturbances were found in a swine model of diffuse TBI,^25^ less is known about the contributions of CBF and energy metabolism alterations in diffuse TBI. The mitochondria play a crucial role in brain energy metabolism and are involved in post-injury oxidative stress with the formation and release of reactive oxygen species (ROS).^26^ While altered mitochondrial respiration following TBI has been observed in different TBI models and age groups,^25,27,28^ studies assessing CBF and mitochondrial function in diffuse TBI are scarce.^29^

Previous data indicate that there are dynamic metabolic impairments in different brain regions following experimental TBI, which depend on time post-injury, the injury model and the injury severity.^30^ The hippocampus is, in view of its important role in executive functions and cognition, of particular interest in TBI. In various rodent models of TBI^31^ including cFPI,^14,15^ working and spatial memory deficits, associated with impaired hippocampal functions, have been observed. How hippocampal changes relate to early alterations in CBF and/or energy metabolism has not been established in the cFPI model.

In the present study, we employed the cFPI model to investigate the potential roles of energy metabolism and CBF in the pathophysiology of diffuse TBI. We assessed CBF using 9.4 T MRI, mitochondrial respiration and ROS production in cortical and hippocampal tissues, as well as hippocampal metabolites using magnetic resonance spectroscopy (^1^H-MRS) at 1- and 7-days post-injury.

## Material and methods

The raw data supporting this study's findings are available from the corresponding author upon reasonable request, and datasets will be made available on Open Data Commons for TBI (ODC-TBI.org)

## Animals

Adult male C57BL/6J mice (pre-injury minimum weight 22 gr, 8–12 weeks old, Taconic, Denmark, n = 105), were housed with *ad libitum* food and water. Mice were randomly selected to either undergo central fluid percussion injury (cFPI) or sham injury using a randomized block design. All procedures were in accordance with the European Union directive (2010/63/EU) and approved by the local ethical committee at Lund University and the Swedish Department for Agriculture (Jordbruksverket, Dnr 4789/2017) and Ethical permit number: M13263-22. The study was conducted in accordance with the ARRIVE guideline 2.0^32^ for the design of the study and reporting of the results.

### Experimental traumatic brain injury

The central (midline) fluid percussion model (cFPI) of diffuse TBI was used as previously described.^33^ The mice were sedated with 4% isoflurane in air, and anesthesia was maintained by 1.2% isoflurane mixed with N_2_O/O_2_ (70:30) delivered via a nosecone for the duration of the surgical preparation. When deeply anesthetized, the mice were placed in a stereotactic frame, the area of the scalp incision infiltrated by bupivacaine (1.25 mg/kg), and a 3.0 mm craniotomy made with a trephine midway between bregma and lambda without damaging the dura mater and the superior sagittal sinus. A plastic connector cap filled with physiological saline (0.9%) was fixed by dental acrylic over the craniotomy and connected to the Luer-Lock on the fluid percussion device filled with distilled water (VCU Biomedical Engineering Facility, Richmond, VA). Pendulum of the fluid percussion device pendulum was released, and a water pressure wave was transmitted into the brain through the craniotomy window. Sham-injured controls were exposed to the same anesthesia and surgical procedure except that the pendulum was not released. The plastic connector was removed after the completion of surgery, the bone plate was put back and the skin was sutured using resorbable sutures. The mice were kept in pre-heated cages and regularly monitored until recovered from anesthesia, then placed into their home cage. Post-operatively, 15 ml paracetamol (24 mg/ml paracetamol) was added to 180 ml drinking water for 3 days.

### Magnetic resonance imaging

Magnetic resonance imaging (MRI) and MR spectroscopy (MRS) were performed to evaluate CBF and energy-related metabolites at day 1 and 7 after cFPI or sham injury. Arterial spin labeling (ASL) was used to determine CBF in the cortex, corpus callosum, hippocampus, thalamus, and cerebellum, and ^1^H MRS was used to evaluate hippocampal metabolites. Animals were anesthetized with O_2_:N_2_O mixed with 2.5% isoflurane in a 1:1 (*v*/*v*) O_2_:N_2_O gas mixture delivered via a mask. The isoflurane concentration was then lowered to 1.5–2% for the duration of the imaging procedure. Respiration was maintained at 70–100 breaths per minute and body temperature kept between 36 and 37 °C with a mouse heating cover from Bruker (Ettlingen, Germany) using circulating warm water. The animals' breathing rate and body temperature were monitored using SA Instruments (Stony Brook, NY, USA) monitoring system throughout the procedure, performed using a 9.4 T preclinical MRI horizontal bore scanner (Agilent, Santa Clara, USA) equipped with Bruker BioSpec AVIII electronics operating with ParaVision 7.0.0 (PV7) and a BGA 12S HP gradient system (Bruker, Ettlingen, Germany) with a maximum gradient strength of 670 mT/m and a rise time of 130 µs. The coils used were a quadrature volume resonator (112/087) for transmission and a mouse brain-phased array coil for the reception in the perfusion measurements. For all other measurements, a transmit-receive quadrature mouse cryo coil was used. All coils were from Bruker.

### Anatomical imaging and hippocampus volume analysis

High-resolution T2-weighted images were obtained with a 2 D RARE sequence. The following parameters were used: TE 44 ms, RARE factor 10, TR 3.4 s, resolution 50 × 50 µm^2^, FOV 18 × 15 mm^2^, and slice thickness 0.5 mm. Thirty-two slices were acquired axially with 6 averages in 10 m 12 s. Hippocampal volumes were automatically calculated by measuring the area of the hippocampus on sequential six 0.5 mm thick T2-weighted coronal slices, which include hippocampal regions throughout the whole anterior-posterior extent by using manually drawn boundaries in OSIRIX MD software (version 12.5.0, Bernex, Switzerland).

### Perfusion imaging

An anatomical reference image for positioning and later masking of the perfusion images were acquired with an axial 2 D RARE. The following parameters were used: TE 33 ms, RARE factor 8, TR 3.5 s, resolution 100 × 100 µm^2^, FOV 16 × 16 mm^2^, and slice thickness 0.5 mm. 32 slices were acquired coronally with 1 average in 2 m 20 s.

This was followed by the acquisition of a 2 D FAIR-RARE scan with slice selective inversion of a 4.5 mm thick slice and 6 inversion times 0.03, 0.5, 1, 3, 5, 9.7 s. The inversion was performed with an adiabatic pulse of sharpness 8 (Bruker terminology), bandwidth of 25 kHz, and length of 4.23 ms. The imaging parameters were TE 25 ms, RARE factor 16, recovery time 10 s, resolution 233 × 234 µm^2^, FOV 17 × 15 mm^2^ and slice thickness 1.5 mm, 1 average, and acquisition time 3 m 58 s. Finally, a FAIR-RARE with slice selective and global inversion was performed with TI = 2 s and 8 averages. The remaining parameters stayed the same. Fourteen mice (sham, n = 6; cFPI, n = 8) were used in this experiment.

### Perfusion data post processing

The perfusion data were evaluated with an own script in a Medical Software for Processing Multi-Parametric Images Pipelines (MP3), a MATLAB plugin.^34^ The fit of the CBF was done according to the following equation, which is a rearrangement of the standard perfusion expression. For more details, please see previously published methods from our group.^35^

$$\mathrm{CBF}=\frac{\lambda \;\Delta M\left(TI\right)\;(R_{1b}-R_{1}^{\mathrm{app}})}{M_{0}2\alpha_{0}\left[e^{-TI\;R_{1}^{\mathrm{app}}}-e^{TI\;R_{1b}}\right]}$$

Here λ is the blood-brain partition coefficient (0.9 mL/g), ΔM the difference in magnetization between selective and global inversion, R_1b_ the measured relaxation rate of blood (0.61 s^−1^),^35,36^ ${\text{R}}_{1}^{\text{app}}$ the relaxation rate in the pixel with selective inversion, M_0_ the equilibrium magnetization measured together with ${\text{R}}_{1}^{\text{app}}$ , α_0_ the inversion efficiency and TI the single inversion time (2 s).

### MR spectroscopy (^1^H MRS)

Spectroscopy was acquired with PV7 according to the following protocol. The acquired T2-weighted images were used for positioning a voxel in the left hippocampus. The voxel dimensions were 2 × 1 × 1.5 mm^3^. Before measuring the spectroscopy data, a field map was acquired. The optimal shim settings based on the field map were calculated over an ellipsoid centered on the voxel and one mm beyond. The full set of higher order shims was used in addition to the linear ones, i.e. 2XY, X2-Y2, Z2, ZX, ZY, Z3, Z4. The contribution of the last two shims was small. This resulted in line widths of 11 ± 1 Hz. All shimming was performed using the standard adjustments procedures of PV7. Localization for spectroscopy was achieved with a STEAM^37^ protocol with TE = 3 ms, TM = 10 ms, and TR = 2.5 s. For the metabolites, twenty blocks of 16 scans were acquired to allow averaging and frequency drift correction. The total acquisition time was 13 m 20 s. An unsuppressed water reference signal to be used as concentration scaling reference was acquired with one block and 16 averages. Pulses shapes calculated by PV7 were used with the sharpness set to 3, resulting in a pulse of 0.28 ms and bandwidth (BW) of 15 kHz. This resulted in a chemical-shift displacement error of 2.7% of the voxel dimensions per ppm. The central frequency for localization was set to 2.35 ppm to minimize the chemical shift displacement error. The STEAM module was preceded by a VAPOR water suppression (WS) sequence with three inserted outer volume suppression (OVS) blocks.^38^ The BW of the WS pulses was 250 Hz. The power amplitudes of the WS pulses were adjusted manually for better results. A half-passage hyperbolic secant pulse with a length of 1.01 ms and BW of 20 kHz was employed for OVS.

### Post-processing MR spectroscopy

The 20 separately acquired blocks of water-suppressed metabolite spectra were summed and frequency aligned to Creatine (Cr) at 3.03 ppm in MATLAB R2020b (The Mathworks, Inc). The signal was also converted to LCModel format. All the subsequent processing was performed in LCModel 6.3.^39^ As Eddy-current compensation was applied already in PV 7, no further correction was applied. The database for fitting contained the following metabolites: alanine (Ala), aspartate (Asp), phosphocholine (PCho), creatine (Cr), phosphocreatine (PCr), gamma-aminobutyric acid (GABA), glutamine (Gln), glutamate (Glu), glutathione (GSH), glycine (Gly), myo-inositol (mI), lactate (Lac), N-acetyl aspartate (NAA), scyllo-inositol (sI), taurine (Tau), ascorbate (Asc), beta-hydroxybutyrate (bHB), glucose (Glc), N-acetyl aspartyl glutamate (NAAG), glycerophosphocholine (GPC), phosphoethanolamine (PE), serine (Ser) and macromolecules (Mac). Only metabolite concentrations estimated with Cramér-Rao lower bound ≤30% were retained for further analysis. Individual correlations between the metabolite concentrations were also checked. This also included NAA+NAAG (tNAA), Ins+Gly, Cr+PCr, and Glu+Gln (Glx). Only metabolites with a correlation ≤0.3 were displayed separately. This meant that the metabolite sums, Ins+Gly, Cr+PCr, Glc+Tau, and GPC+PC (tCho) had to be employed. Also, Glu+Gln and NAA+NAAG are displayed even though this is not following from the results of the correlation analysis.

### Mitochondrial high-resolution respirometry (HRR)

The mice were anesthetized using sodium pentobarbital (60 mg/ml), and when deeply sedated brains were rapidly removed from the skull and placed in ice-cold mitochondrial respiration medium MiR05 (0.5 mM EGTA, 3 mM MgCl_2_, 60 mM K-lactobionate, 20 mM taurine, 10 mM KH_2_PO_4_, 20 mM HEPES, 110 mM sucrose, 1 g/l BSA, pH 7.2).^40^ Tissue from the cortex and hippocampus bilaterally was weighed and manually homogenized in a Teflon-glass potter homogenizer to a concentration of 1 mg wet weight tissue/10 μl MiR05.^40^ All steps were performed on ice and all buffer solutions were ice-cold.

Mitochondrial respiration was measured using a high-resolution Oroboros Oxygraph-2k (Oroboros Instruments, Innsbruck, Austria), as previously published.^41^ Samples were run simultaneously with a volume of 2 ml each and with a constant temperature of 37 °C. Experiments were started by adding 20 μl of brain homogenate (2 mg wet-weight brain tissue) into chambers prefilled with MiR05 to a final concentration of 1 mg/ml. Oxygen consumption and oxygen flux were monitored and recorded using DatLab 7.4 software (Oroboros Instruments, Innsbruck, Austria).

A substrate, uncoupler, inhibitor titration (SUIT) protocol with consecutive additions was utilized to acquire a detailed representation of mitochondrial respiration. The respiratory capacity under oxidative phosphorylation (OXPHOS) with electron flow through complex I (CI) alone (OXPHOS_CI_) as well as the maximum convergent electron input through the Q-junction (OXPHOS_CI + II_)^40^ were assessed, and also the maximal non-phosphorylating respiration (termed electron transport system, ETS) with both CI and CII substrates (ETS_CI+CII_) and CII alone (ETS_CII_) were recorded.

First, the homogenates were stabilized at basal respiration without exogenous substrates in MiR05. Thereafter, malate (5 mM) and pyruvate (5 mM) were added followed by ADP (1 mM) and glutamate (5 mM) (OXPHOS_CI_) showing respiration driven by the NADH-related substrates. Subsequently, succinate (10 mM) was added stimulating maximal OXPHOS capacity by convergent input through CI and CII (OXPHOS_CI+CII_). Oxidative phosphorylation was blocked by oligomycin, an ATP-synthase inhibitor, inducing LEAK respiration (LEAK_CI+CII_), i.e. mitochondrial respiration independent of ATP production. The maximal convergent respiratory capacity of the electron transport system (ETS_CI+CII_) was then evaluated by titrating the protonophore, carbonyl cyanide p-(trifluoromethoxy) phenylhydrazone (FCCP) until no further increase in respiration was detected. To measure the ETS capacity supported by succinate alone through CII (ETS_CII_) rotenone was added to inhibit CI. Finally, electron flow through the ETS was blocked by the addition of the complex III (CIII) inhibitor antimycin-A (1 μg/ml) revealing the residual oxygen consumption not related to the ETS or OXPHOS. This value was subtracted from the different respiratory states in the final analysis. Respiratory control ratios (RCR) were calculated for both maximal capacities of OXPHOS and ETS by dividing the respective rate by LEAK respiration rate. The relationship between maximum OXPHOS convergent respiration (OXPHOS_CI+CII_) and maximum convergent ETS was evaluated by calculating a ratio OXPHOS_CI+CII_/ETS_CI+CII_ (OXPHOS/ETS), to evaluate any limitation on the respiratory chain at level of the ATP synthase.

### Reactive oxygen species (ROS) fluorometry measurements

ROS production was measured simultaneous to HRR using the O2k-Fluo LED2 add on fluorometry module (Oroboros Instruments, Innsbruck, Austria). Before addition of the above-mentioned substrates in the SUIT protocol as specified for HRR, 2 µL Amplex Red (10 µM), 8 µL horseradish peroxidase (250 U/mL) and 2 µL superoxide dismutase (10 kU/mL) was added. Superoxide dismutase converts all superoxide to H_2_O_2_, horseradish peroxidase facilitates Amplex Red to bind with H_2_O_2_ to produce the fluorophore resorufin which intensity is a quantifiable measure of ROS production. H_2_O_2_ flux was corrected for the slope determined in the presence of homogenate, and fluorescence signals were calibrated using H_2_O_2_ titrations at the routine state (post homogenate addition).

### Caspase -3 immunofluorescence staining

Immunofluorescence staining was performed using an anti- rabbit Caspase-3 antibody (ThermoFisher PA577887; dilution 1:1000). followed by Cy3 (working dilution 1:500) fluorophore-conjugated secondary antibodies (Jackson Immunoresearch, US). The stainings were visualized on 40× magnification in Leica SP8 laser-scanning confocal microscope (Germany). For additional details see Methods to Suppl. Fig. 1.

### Statistical analysis

Statistical analysis of high-resolution respirometry experiments was performed using 2-way ANOVA with Tukey’s multiple comparisons test between sham and cFPI for each parameter at each time point. Significance was set at *P* < 0.05.

The obtained metabolite concentrations by ^1^H MRS, CBF-values and hippocampal volume data were subjected to permutation test analysis in R.4.4.3 (https://www.r-project.org/). This analysis can be applied in an explorative study such as our with a small sample size.^42–44^ Accurate estimation of multiple comparison errors is difficult on small amounts of samples and bigger amounts of samples are neither ethical according to the 3 R principle, nor practical. In the permutation analysis we tested the null hypothesis of the CBF, metabolite concentrations or hippocampal volumes being the same at the two time points or under the conditions Sham or cFPI. The test statistics were the absolute difference between the means for the compared groups. A number of 10 000 permutations were performed and the resulting p-value gives the probability of obtaining the observed difference if CBF, metabolite concentrations or hippocampal volumes were the same.

## Results

We included 105 mice, of which 15 were excluded from analysis due to death at impact or post-surgical complications (dural tear, too long apnea), leaving 90 animals for further analysis. The quality of the pressure pulse was verified in all cases, the mean pressure was 2.18 ± 0.22 atm, and the apnea induced by cFPI had a mean duration of 50 ± 2.3 seconds.

### Diffuse axonal injury induces widespread alterations of cerebral blood flow throughout the injured brain

**Figure 1.**
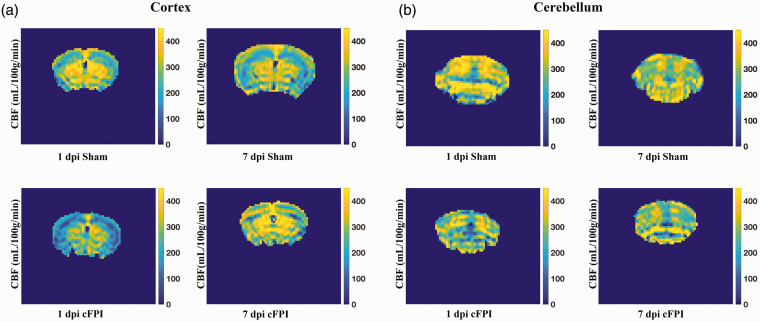
Assessment of cerebral blood flow (CBF) following traumatic axonal injury in the mouse Magnetic resonance imaging was performed to evaluate CBF at day 1 and 7 after injury onset. Representative arterial spin labeling (ASL) MRI images visualizing perfusion measured in ml/100g/min in the cortex (a) and cerebellum (b).

Reduced CBF can impair brain function and although it rarely reaching ischemic levels following TBI,^45,46^ it often coincides with periods of increased metabolic energy demand. CBF was assessed in the bilateral cortex and hippocampi, corpus callosum, thalamus and cerebellum in cFPI and sham groups by repeating ASL measurements at 1- and 7-days post-injury (dpi) following cFPI, representing images are presented in Figure 1(a) and (b). Fourteen mice (sham, n = 6; cFPI, n = 8) that survived to 7 dpi were included in this longitudinal assessment.

**Figure 2.**
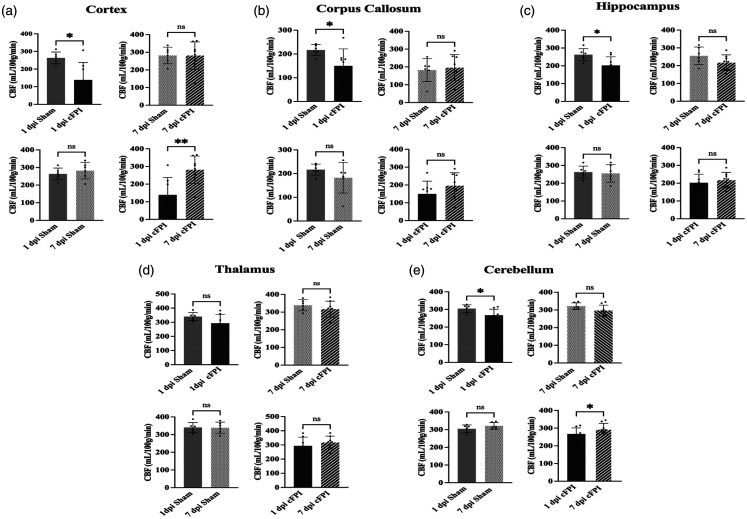
Traumatic axonal injury in mice causes an early widespread reduction in cerebral blood flow (CBF) assessed by 9.4 T MRI arterial spin labeling (a) Cortical CBF was reduced by cFPI (n = 8) when compared to Sham (n = 6) at 1 dpi (p < 0.05), reductions normalized by 7 dpi (p < 0.01) (b) Similarly in the corpus callosum, CBF was lower in cFPI mice (n = 8) than in Sham (n = 6) mice at 1 dpi (p < 0.05) but not at 7 dpi (c) Hippocampal CBF was reduced by cFPI (n = 8) when compared to Sham (n = 6) at 1 dpi (p < 0.05), normalized at 7 dpi (p > 0.05) (d) Thalamic blood flow was not altered following cFPI at any evaluated time point (p > 0.05) and (e) CBF in the cerebellum was reduced in cFPI (n = 8) when compared to Sham (n = 6) at 1 dpi (p < 0.05), although not at 7 dpi (p < 0.01). Individual data points are shown, bar graphs shown as mean ± SD. (*p < 0.05, **p < 0.01, ns = non-significant).

Analysis of the impact-adjacent cortical regions revealed significantly reduced CBF in the cFPI group compared to sham controls at 1 dpi (p < 0.05). By 7 dpi, CBF in the cFPI group had normalized to sham control levels (p > 0.05), and the cFPI group had reduced CBF at 1 dpi when compared to 7 dpi (p < 0.01). In the sham-injured group, CBF was similar at 1 and 7 dpi (p > 0.05) (Figure 2(a)). In the corpus callosum, CBF was decreased in the cFPI group at 1 dpi (p < 0.05), but not at day 7 post-injury when compared to sham-injured controls (p > 0.05). CBF levels were not significantly altered between 1 and 7 dpi in either the cFPI or sham groups (p > 0.05) (Figure 2(b)). The CBF was bilaterally assessed in the hippocampi, revealing a significant reduction in the cFPI group compared to the sham-injured controls at 1 dpi (p < 0.05). However, this difference was no longer observed at 7 dpi. Additionally, there were no differences in hippocampal CBF between 1 dpi and 7 dpi either in the sham or cFPI groups (p > 0.05) (Figure 2(c)). In the thalamus, the CBF was similar between sham-injured and cFPI animals at both time points (p > 0.05) (Figure 2(d)). A recent study from our group showed that traumatic axonal injury disrupts myelin integrity of the cerebellum;^47,48^ therefore, we analyzed the CBF of the cerebellum and found it to be significantly decreased by 1 dpi 1 in the cFPI group when compared to controls (p < 0.05). At 7 dpi it was similar between the groups (p > 0.05). The CBF in sham-injured controls was similar at 1 dpi and 7 dpi (p > 0.05) while the cerebellar CBF was higher at 7 dpi when compared to 1 dpi in the cFPI group (p < 0.05) (Figure 2(e)).

### Neither mitochondrial respiration nor ROS generation was altered by TBI

In separate experiments, 32 mice were used to evaluate mitochondrial respiration and ROS generation. The mice were divided into four independent groups as 1 dpi Sham (n = 8), 1 dpi cFPI (n = 10), 7 dpi Sham (n = 7), 7 dpi TBI (n = 7).

**Figure 3.**
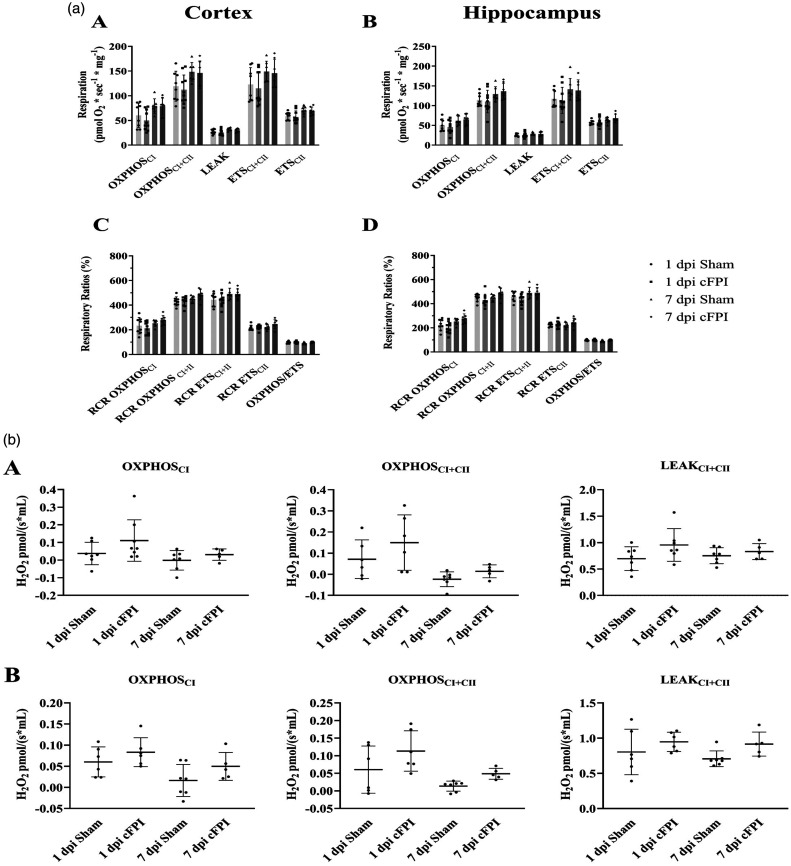
(a) Mitochondrial respiration in cortex and hippocampus at 1 and 7 dpi following cFPI or sham injury. Here, absolute values of mitochondrial respiration in homogenized brain tissue of mice subject to cFPI and sham are shown in (A) cortex, and (B) hippocampus, as well as RCR of each respiratory state and a ratio of phosphorylating and non-phosphorylating maximum mitochondrial respiration in (C) cortex and (D) hippocampus. Data were analyzed using a 2-way ANOVA with Tukey’s multiple comparisons test, separately for Cortex and Hippocampus. Significance was set at *P* < 0.05. n = 8 for 1 dpi Sham, n = 10 for 1 dpi cFPI, n = 7 for 7 dpi Sham and n = 7 for 7 dpi cFPI. RCR, respiratory control ratio; dpi, days post injury; OXPHOS, oxidative phosphorylation; CI, complex I; CII, complex II. Data presented as Mean ± SD and (b) Reactive oxygen species (ROS) generation in cortex and hippocampus at 1 and 7 dpi following cFPI or sham injury in (A) cortex, and (B) hippocampus. Two-way ANOVA with Tukey’s multiple comparisons test was performed separately for the Cortex and Hippocampus (P < 0.05). Sample sizes were: Cortex; 1 dpi Sham (n = 7), 1 dpi cFPI (n = 8), 7 dpi Sham (n = 7), 7 dpi cFPI (n = 5); Hippocampus; 1 dpi Sham (n = 6), 1 dpi cFPI (n = 6), 7 dpi Sham (n = 7), 7 dpi cFPI (n = 5); OXPHOS, oxidative phosphorylation; CI, complex I; CII, complex II. Data presented as Mean ± SD.

Tissue from both cortex and hippocampus was analyzed at both time points (Figure 3(a)). The absolute levels of mitochondrial respiration was compared between cFPI and sham for OXPHOS_CI_ (oxidative phosphorylation of NADH-linked substrates), OXPHOS_CI+II_ (convergent oxidative phosphorylation of both NADH and FADH_2_-related substrates), LEAK (respiration not related to ATP-production), ETS_CI+CII_ (the maximal induced convergent respiratory capacity of the electron transport system) and that relating to CII alone, ETS_CII_, with no significant difference detected (all p > 0.05). Additionally, to further control for potential influence of variation between experiments, respiratory control ratios (RCRs) were calculated and compared for each state (/LEAK), with no significant difference detected. Also, the ratio of phosphorylating and non-phosphorylating maximum mitochondrial respiration was analyzed for each tissue and time point without any significant difference between sham and cFPI groups, neither at 1 dpi nor at 7 dpi. The corresponding comparisons for ROS generation between treatment groups and time points, likewise, did not demonstrate any statistically significant differences (p > 0.05) (Figure 3(b)).

### Delayed alterations in hippocampal metabolites following widespread traumatic axonal injury, evaluated by ^1^H MRS

**Figure 4.**
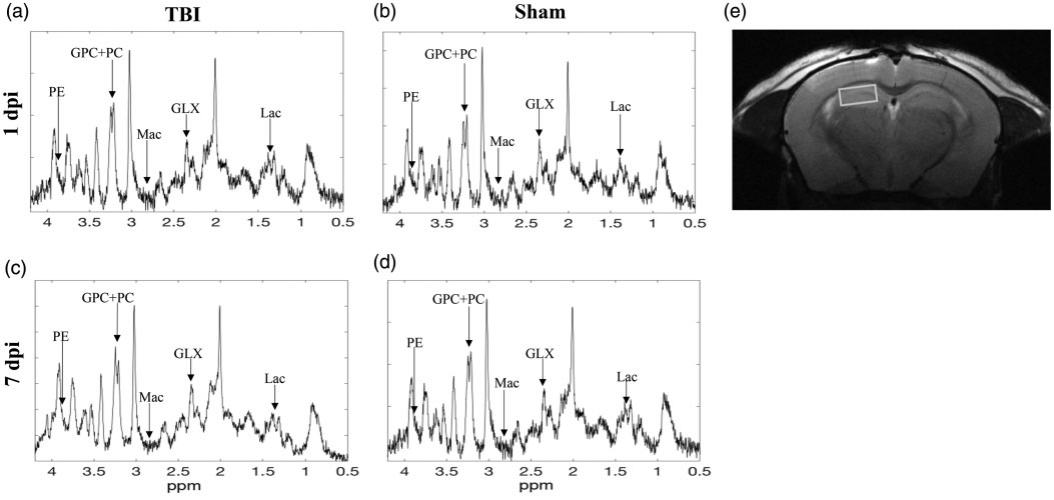
Representative ^1^H MRS spectra of TBI (left) and sham (right) at the time at 1 dpi days (a and b) (top) and (c and d) 7 dpi (bottom). The spectra have been line broadened with 2 Hz. (e) The location of voxel in the hippocampus. The units on the vertical axis are arbitrary.

**Figure 5.**
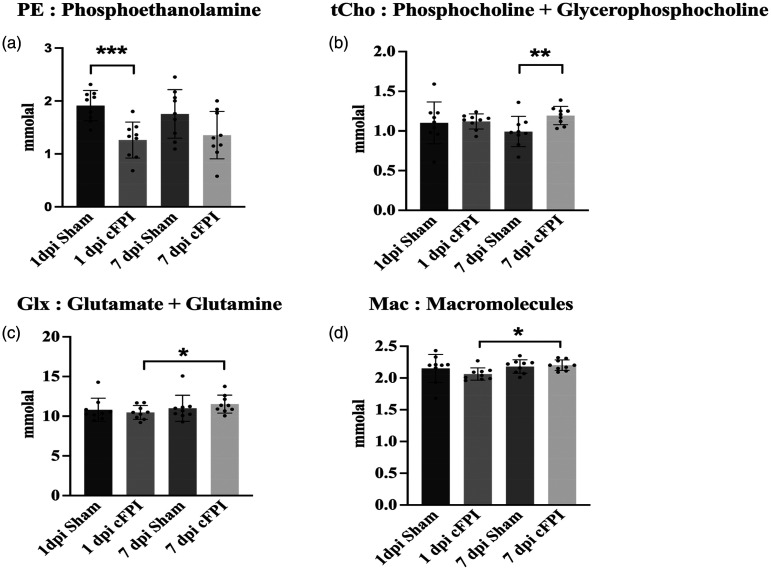
^1^H MRS data results based on paired and unpaired t-test. (a) Phosphoethanolamine (PE) level significantly decreased between 1 dpi Sham and 1 dpi sham groups (permutation test, p < 0.001) (b) Phosphocholine and Glycerophospocholine (tCho) significantly increased between 7 dpi Sham and cFPI groups (permutation test, p < 0.01) (c) Glutamate and Glutamine (Glx) levels significantly increase from 1 dpi to 7 dpi cFPI (permutation test, p < 0.05) and (d) Macromolecule levels significantly increased from 1 dpi cFPI to 7 dpi cFPI (permutation test, p < 0.01). Data presented as Mean ± SD.

We analyzed post-injury changes in the hippocampus using ^1^H MRS data (Figures 4 and 5), evaluated statistically with a permutation test (Table 1).

**Table 1.** Hippocampal metabolites with mean and standard deviation (column to the right) in mmolal.

Metabolite	1 dpi cFPI (1)		7 dpi cFPI (2)		1 dpi Sham (3)		7 dpi Sham (4)		1 vs 2	3 vs 4	1 vs 3	2 vs 4
Ala	1.13	0.69	0.91	0.60	1.41	0.64	0.90	0.54	0.47	0.09	0.39	0.98
Asp	0.19	0.57	0.34	0.68	0.37	0.74	0.18	0.53	1.00	0.47	1.00	1.00
GABA	1.29	0.51	1.41	0.34	1.38	0.17	1.42	0.20	0.61	0.71	0.77	0.96
Gln	3.14	0.72	3.48	0.63	2.92	1.25	3.07	1.40	0.30	0.72	0.67	0.49
Glu	7.33	1.26	8.04	0.75	7.90	0.80	7.94	0.57	0.17	0.91	0.28	0.75
GSH	1.55	0.20	1.42	0.25	1.57	0.20	1.57	0.25	0.23	1.00	0.91	0.23
Lac	3.61	1.67	2.90	1.10	3.61	1.10	2.64	0.75	0.30	**0.04**	0.99	0.57
NAA	6.53	0.65	6.69	0.51	6.75	0.76	7.17	0.55	0.59	0.20	0.51	0.07
Asc	2.89	0.84	2.39	0.99	3.15	0.96	2.65	0.64	0.29	0.23	0.56	0.57
NAAG	0.21	0.32	0.17	0.26	0.19	0.29	0.12	0.23	0.70	0.43	0.85	0.72
PE	0.49	0.75	1.03	0.83	1.91	0.29	1.50	0.90	0.18	0.25	**0.0003**	0.26
Mac	2.06	0.10	2.20	0.08	2.15	0.22	2.18	0.11	**0.007**	0.75	0.30	0.64
NAA+NAAG	7.04	0.64	7.10	0.51	7.23	0.78	7.58	0.52	0.85	0.30	0.58	0.06
Ins+Gly	6.50	0.94	6.59	1.19	6.68	0.97	7.03	1.46	0.86	0.56	0.71	0.49
Cr+PCr	9.44	0.58	9.25	0.90	9.53	0.89	9.24	0.49	0.61	0.42	0.80	0.98
Glu+Gln	10.47	0.87	11.53	1.13	10.81	1.45	11.01	1.64	**0.04**	0.76	0.61	0.46
Glc+Tau	12.59	1.73	12.72	1.83	12.65	1.77	12.39	1.45	0.88	0.74	0.94	0.69
GPC+PC	1.12	0.10	1.19	0.12	1.10	0.26	0.95	0.20	0.15	0.20	0.86	**0.007**

Group comparison of the difference in means was performed by permutation analysis in R and the result signifies the probability of obtaining the observed absolute difference in means if the compared groups if the metabolite concentrations were the same at both compared time points or conditions. Bold values indicate significance differences of P-values < 0.05. Dpi: days post-injury; cFPI: central (midline) fluid percussion. Alanine (Ala), aspartate (Asp), gamma-aminobutyric acid (GABA), glutamine (Gln), glutamate (Glu), glutathione (GSH), Lactate (Lac), N-acetyl aspartate (NAA), ascorbate (Asc), N-acetyl aspartyl glutamate (NAAG), phosphoethanolamine (PE), macromolecules (Mac), glycine (Gly), creatine (Cr), phosphocreatine (PCr), glucose (Glc), taurine (Tau), and glycerophosphocholine (GPC), and. Only metabolites estimated with Cramér-Rao lower bound ≤ 30% were retained for further analysis. Individual correlations between the metabolite concentrations were also evaluated. This also included NAA+NAAG (tNAA), Ins+Gly, Cr+PCr, and Glu+Gln (Glx). Only metabolites with a correlation ≤ 0.3 were displayed separately. This meant that the metabolite sums, Ins+Gly, Cr+PCr, Glc+Tau, and GPC+PC (tCho) had to be used.

Lactate (Lac) is produced in anaerobic conditions to sustain glycolysis when NADH cannot be oxidized at mitochondrial complex I. Lactate can act as a neuronal energy reserve and can be increased by ischemia, hypoxia, and macrophage infiltration.^30,49^ Lac level did not change significantly following cFPI indicating that the reduced CBF changes in the hippocampus were insufficient in producing an energy metabolic disturbance (Table 1). Total NAA (tNAA), expressed as combination of NAA and NAAG, gives information regarding neural loss and/or dysfunction. While tNAA has been shown to decrease following TBI, we did not observe a significant difference between our groups in neither early (1 dpi) or delayed time point (7 dpi) (Table 1), confirming the results of our previous clinical study at long-term after TBI.^50^

Phosphoethanolamine (PE) is involved in brain phospholipid metabolism,^51^ and was significantly lower at 1 dpi cFPI (0.49 ± 0.75 mmolal), when compared to the sham group at the same time point (1.91 ± 0.29 mmolal, p = 0.003) (Figure 5(a); Table 1).

Choline-containing compounds are a substantial part of cell membranes and are necessary for the synthesis of the neurotransmitter acetylcholine. Changes in total choline (tCho), which is phosphocholine (PC) and glycerophosphocholine (GPC), and free choline reflect cell membrane integrity.^52^ Choline-including metabolites are not soluble under normal conditions, therefore increased choline level are considered as a marker of membrane disruption detectable by MRS.^53^ Our results show that GPC + PC was significantly higher in the cFPI group at 7 dpi (1.19 ± 0.12 mmolal) when compared to the 7 dpi sham group (0.95 ± 0.20 mmolal, p = 0.007), which indicates cell membrane disruptions (Figure 5(b)) (Table 1).

Glutamate (Glu), synthesized from Glutamine (Gln), is the most abundant excitatory neurotransmitter. Glu is involved in a large number of metabolic events, and excessive release upon injury may result in cellular excitotoxicity which is a common secondary injury mechanism following TBI.^54^ Glutamate (Glu) + Glutamine (Gln) are also commonly expressed together (Glx) since they are highly overlapped on the ^1^H MRS spectrum. Our data indicate that the Glu + Gln levels were significantly lower in brain-injured animals at 1 dpi (10.47 ± 0.87 mmolal) when compared to the cFPI group at 7 dpi (11.53 ± 1.13 mmolal; P = 0.040) (Figure 5(c): Table 1).

Macromolecules (Mac) are high molecular-weight compounds.^55^ The Mac signal may arise from different amino acids within cytosolic proteins, and mobile proteins/peptides as well as post-translational modifications of proteins, and finally signal from mobile lipids and necrotic tissue that may overlap with the Mac signal on *in vivo* MRS. We found that Mac levels increased from 1 dpi cFPI (2.06 ± 0.09 mmolal) to 7 dpi cFPI (2.20 ± 0.08 mmolal, p = 0.013) (Figure 5D) (Table 1).

### Hippocampal volumes and cell death is not altered following diffuse TBI

**Figure 6.**
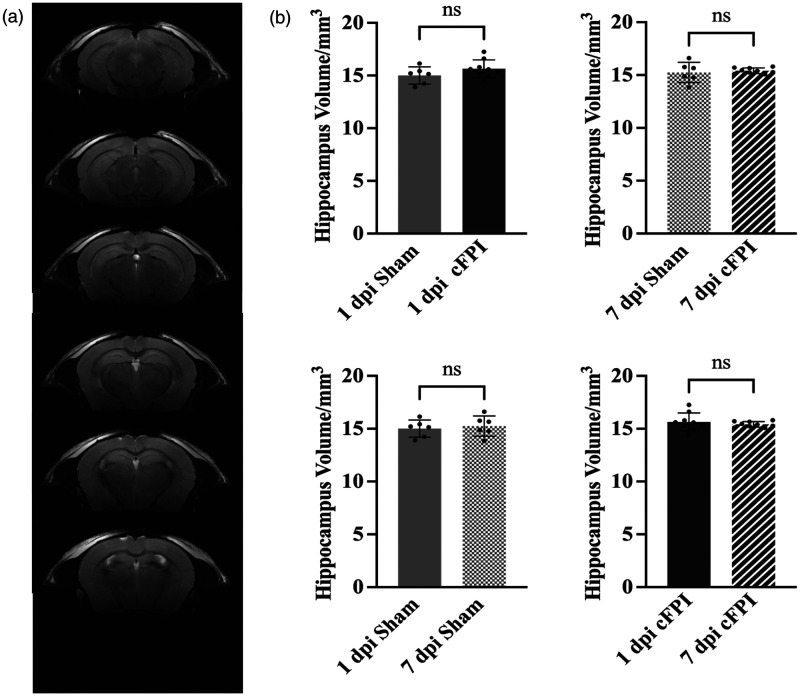
Hippocampal volumes did not change following TBI (a) The representative T_2_-weighted images of hippocampal regions (coordinates were on bregma from −1.06 mm to −3.28 mm) used in the data analysis. Each slice thickness is 0.5 mm and (b) There were no significant differences between the groups (p > 0.05). Hippocampal volume analysis was performed by OsiriX MD (Version 12.5.0). Data presented as Mean ± SD.

Our ^1^H MRS, mitochondrial and CBF analyses could potentially be influenced by extensive structural damage to e.g. the hippocampus. Therefore, given the impaired hippocampal function commonly observed in this diffuse TBI model, we evaluated hippocampal volumes using MRI and and evaluated apoptotic cell death by immunohistochemistry. The T2-weighted MRI sequences were used to evaluate hippocampal volumes, revealing no significant differences between sham-injured and cFPI groups at either 1 dpi or 7 dpi (p > 0.05) (Figure 6).

To access, cell death, we performed immunohistochemistry staining for caspase-3, a marker for apoptosis. Since merely a few activated caspase-3 positive cells were observed in other regions, we focused our analysis on the dentate gyrus of the hippocampus.^56^ No differences between the sham-injured and cFPI groups were observed (Suppl. Figure 1). Thus, we observed merely limited cell death observed in the hippocampus in our model, similar to in our previous report.^57^ Twenty-four mice were used in this experiment; 1 dpi Sham (n = 6), 1 dpi cFPI (n = 6), 7 dpi Sham (n = 6), 7 dpi TBI (n = 6),

## Discussion

In the present study, we investigated the early dynamics of cerebral blood flow (CBF) and energy metabolism in a mice model of diffuse TBI. The injury severity was targeted to be mild-moderate consistent with the histological consequences, and some acute mortality, observed in this model. We observed that cFPI induced widespread although transient reductions in CBF across both white and gray matter regions as well as in the cerebellum. However, neither mitochondrial respiration nor ROS generation was altered by the brain injury. Moreover, our data suggest that transient CBF reduction did not result in structural injury to the hippocampus as there were no alterations of hippocampal volume, nor signs of overt cell death. Using ^1^H MRS, we evaluated changes of hippocampal metabolites and did not observe any change in the hippocampal lactate levels between brain- and sham injured controls supporting the notion that this TBI model does not result in overt energy disturbance. In contrast, we observed reductions in phosphoethanolamine, related to phospholipid metabolism, at 1 dpi and more pronounced delayed (7 dpi) changes in other key metabolites. These included delayed reductions in Choline-containing compounds, integral components of cell membranes. Moreover, the 7 dpi increase in Glutamate (Glu) + Glutamine (Gln) may indicate delayed excitotoxicity to the hippocampus. We cannot exclude that these changes were initiated by the early decreased CBF although, regardless, these alterations of factors related to excitotoxicity and impaired membrane integrity may be important contributors to the consistently observed cognitive dysfunction in this TBI model.^15^

Injury to the cerebral vasculature and impaired CBF are increasingly recognized as key contributors to TBI-related neurologic dysfunctions^58,59^ potentially driven by mechanism such as loss of pericytes and endothelial disruptions.^60^ In clinical TBI, CBF changes are highly dynamic with early CBF reduction, however rarely reaching ischemic levels. In contrast, in TBI patients who die from their injury ischemic lesions are commonly observed.^61^ In our mouse model of widespread traumatic axonal injury (cFPI), we observed significantly reduced CBF at one-day post-injury in several brain regions such as the cortex, corpus callosum, hippocampus, and cerebellum. Notably, these reductions were transient and returned to baseline levels at 7 dpi. Such dynamic changes in the cerebrovascular function have previously been observed in other TBI models^58,62^ and in support of clinical observations.^63^ Next, we aimed to investigate whether this transient yet widespread reduction of CBF translated into a potentially detrimental energy metabolic disturbance.

Loss of mitochondrial membrane potential as well as impairments of ETS and OXPHOS capacity are commonly observed in different TBI models.^25,27,28,64,65^ In particular, mitochondrial respiration in the cortex and hippocampus is impaired in rodent models from as early as 1 dpi and can persist up to 14 dpi.^66,67^ However, in our present data we did not observe any significant difference related neither to mitochondrial respiration nor ROS generation between sham and cFPI group at any of the time points. While there was non-significant trend towards higher ROS generation at 1 dpi in both the cortex and hippocampus, this did not persist until 7 dpi.

There are many different TBI models, selected to mimic the vast heterogeneity of clinical TBI. Here, we used the well-characterized cFPI model that shows persistent physiological and histological consequences.^33,68^ The pathophysiological mechanisms seem similar between the humans and rodents on the physiological and molecular level arguing for translational value of this model despite the obvious differences in terms of complexity, overall brain structure, and tissue organization.^16,17^ The focal controlled cortical impact (CCI) or lateral fluid percussion (lFPI) model shows more cell death and focal tissue disruption when compared to the cFPI model. Similar to our results, a previous study using lFPI in the rat did not find any significant differences in mitochondrial respiration rates in cortical and hippocampal brain regions.^69^ Alterations of ATP concentration and cerebral metabolism might, however, occur without uncoupling respiration following fluid percussion injury.^70–72^ Through reduced CBF, and the associated reduction in oxygen delivery, TBI may result in a shift toward anaerobic glycolysis and increased brain lactate when NADH cannot be oxidized in the mitochondrial electron transport system (ETS).^71,72^ In our present study there was no difference in lactate levels between sham- and cFPI groups, suggesting that the reduced CBF in the hippocampus was insufficient in causing a shift towards anaerobic glycolysis due to insufficient oxygen supply. Mitochondrial respiratory dysfunction may also result in an increase in lactate levels independent of oxygen levels. There was no difference in lactate levels measured with MRS between sham- and cFPI groups, in line with the data from mitochondrial respiration measured *ex vivo*, and these data argue there is neither a metabolic energy disturbance in this model, nor a lack of oxygen delivery. The cFPI model does thus not induce a dysfunction of the ETS at the mitochondrial level related neither to respiration and ATP production nor ROS generation.

Magnetic resonance spectroscopy (^1^H MRS) enables a non-invasive estimate of the energy metabolic situation in a selected area. Our ^1^H MRS finding indicates that the levels of N-acetyl aspartate (NAA) + N-acetylaspartyl-glutamate (NAAG) level were not altered following cFPI. When compared to day 1, lactate levels at day 7 were lower and similar in both the cFPI and sham groups. While the difference reached statistical significance only for sham the data would rather be interpreted as anesthesia and the surgical exposure having the possibility to increase brain lactate level, not exacerbated by the cFPI brain injury and these data emphasizes the need to use surgically exposed controls as controls in experiments. By MRS, we found a significant reduction in phosphocholine (PC) and glycerophosphocholine (GPC) at 7 dpi following the brain injury. In the present traumatic axonal injury model, our data suggests a potential membrane breakdown and/or demyelination in the hippocampus. Glutamate and glutamine levels significantly increased from 1 dpi to 7 dpi in the brain-injured group. Increased levels of these excitatory compounds may plausibly contribute to a delayed and potentially persistently increased excitotoxicity, and contribute to neuronal vulnerability, aberrant plasticity and cognitive dysfunction.^73–75^ Moreover, ongoing glutamate release has been interpreted as indicating viable tissue,^76^ supported by the limited cell death observed in our present study. Finally, many different factors could contribute to the macromolecule (Mac) signal (e.g., amino acids, mobile lipids, and mobile proteins) and other methods are needed to establish what factors are most relevant in TBI pathophysiology.

Apoptotic cell death has been observed in both pre-clinical^77^ and clinical TBI.^78^ It commonly occurs in the cortex and hippocampus from 1 dpi up to 2 weeks post injury, based on the severity of injury and model of TBI.^56,77^ Our data did not indicate extensive apoptotic cell death in the hippocampus at either 1 dpi or 7 dpi, in contrast to in focal TBI models where marked apoptotic cell death is commonly observed.^79^ We also assessed hippocampus volumes following the cFPI by using T2-weighted image analysis and found that the cFPI model did not result in reduced hippocampal volumes.

TBI is associated with complex multifaceted secondary injury mechanisms which are not limited to CBF, mitochondrial impairments, and metabolic imbalances. While many of the secondary injury mechanisms of TBI remain unknown, both the early and delayed changes observed by MRS, and the early CBF changes, are likely contributing. In our experiments, we did not perform any behavior tests to evaluate cognitive functions in the cFPI mice in view of the short survival time points. However, persistent behavioral deficits have been repeatedly shown in the cFPI model in many studies from us and others.^14^ Recently, reduced CBF correlated to persistent cognitive dysfunction, sustained up to 6 months post-injury.^15^ One limitation of the present study is that we did not use longer post-injury time points, which was beyond the scope of a single study. In addition, while we aimed at an injury-related mortality of 10% an even higher injury severity level could have resulted in a more profound energy metabolic disturbance.

In summary, we observed an early, widespread CBF reduction post-injury that normalized by 7 dpi. This reduction in CBF was not associated with altered mitochondrial respiration, ROS formation, apoptotic cell death, or reduced hippocampus volumes. Instead, delayed injury mechanisms related to excitotoxicity and membrane disruptions were observed. These results suggest a complex pathophysiology of traumatic axonal injury, to which a transient reduction of CBF may contribute while other, delayed mechanisms may be additional contributors to the observed hippocampal pathophysiology post-TBI.

## Supplemental Material

sj-pdf-1-jcb-10.1177_0271678X251364136 - Supplemental material for Diffuse traumatic brain injury in mice is associated with a transient mismatch of cerebral blood flow and energy metabolism Supplemental material, sj-pdf-1-jcb-10.1177_0271678X251364136 for Diffuse traumatic brain injury in mice is associated with a transient mismatch of cerebral blood flow and energy metabolism by Sertan Arkan, Michael Gottschalk, Saema Ansar, Jesper Peter Bömers, Johannes Ehinger, Eskil Elmér, Imen Chamkha, Michael Karlsson and Niklas Marklund in Journal of Cerebral Blood Flow & Metabolism
