# Supplementary material for: Diffuse traumatic brain injury in mice is associated with a transient mismatch of cerebral blood flow and energy metabolism
Source: J Cereb Blood Flow Metab. 2025 Aug 13;46(1):118–33. doi: 10.1177/0271678X251364136 (PMC12350319; doi:10.1177/0271678X251364136)
Supplement: Supplementary file 1 [file sj-pdf-1-jcb-10.1177_0271678X251364136.pdf]

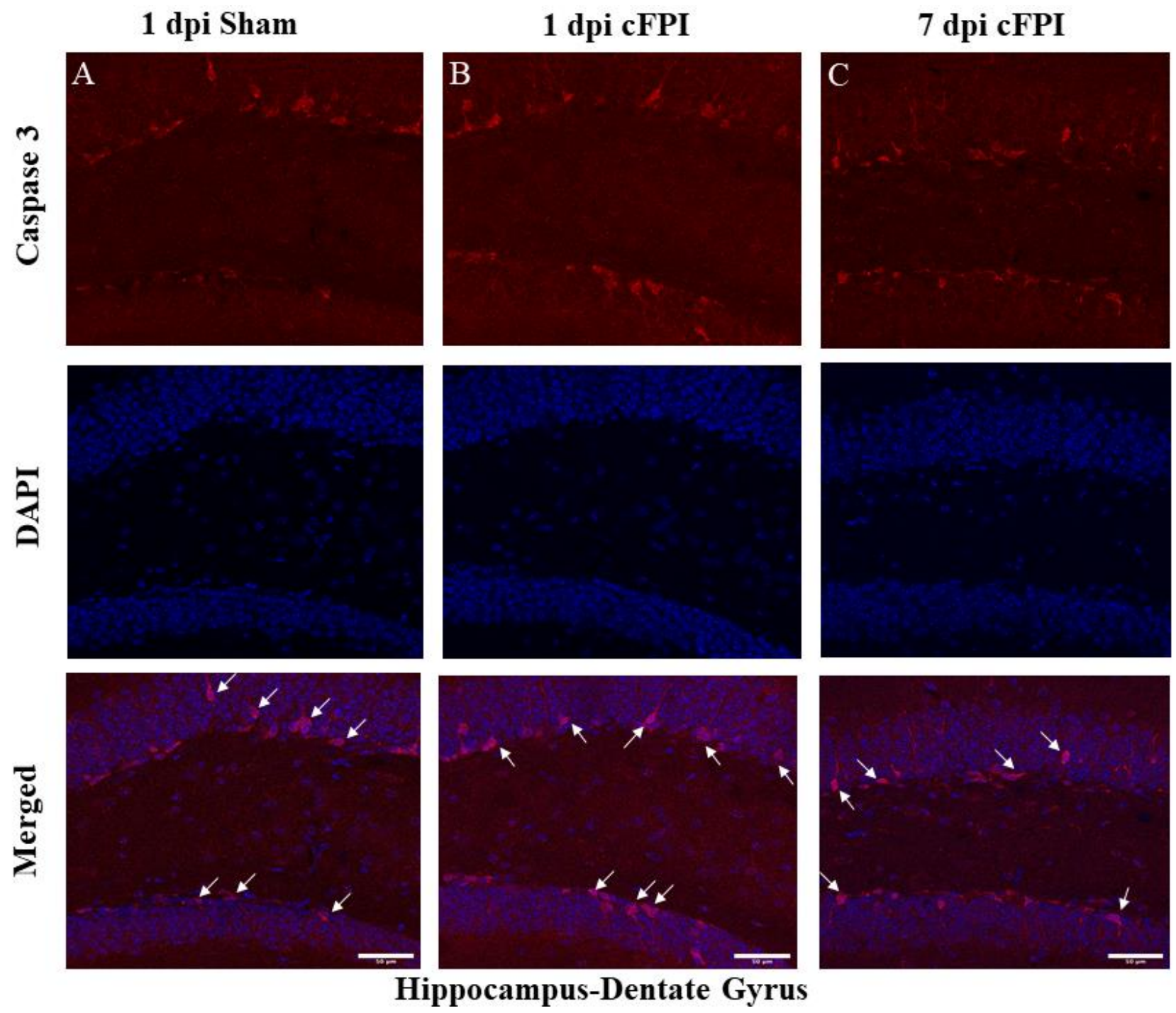

Supplemental Figure 1: Traumatic axonal injury using the cFPI model did not result in an increased number of apoptotic cell death in the dentate gyrus of the hippocampus. Representative confocal images of Caspase-3 staining. DAPI is a nucleus marker, and Cy3 was used to visualize activated Caspase-3 positive cells A) 1 dpi sham B) 1 dpi cFPI and C) 7 dpi cFPI (Data is not shown) Arrows indicate Caspase-3 expressing cells. Scale bar = 50 um

Methods to Supplemental Figure 1

Immunofluorescence staining was performed using an anti- rabbit Caspase-3 antibody (ThermoFisher PA577887; dilution 1:1000). Free-floating sections were washed thrice with PBS from an anti-freeze solution. The sections were then incubated in 0.05% Triton X-100 (Sigma Aldrich) in PBS containing 10% of normal donkey serum (NDS) (Jackson ImmunoResearch, US) for 1h. The sections were subsequently incubated with primary antibody, diluted with 3% NDS in PBS,overnight at 4 °C. Then, sections were rinsed with 0.05% PBS-Triton X-100 for 5 min, then incubated with Cy3 (working dilution 1:500) fluorophore-conjugated secondary antibodies (Jackson Immunoresearch, US). Following

washing steps with PBS, sections were stained by DAPI (dilution 1:2000) for 10 min, three times rinsed with PBS, and mounted on positive charged super frost plus glass slides (ThermoScientific, US). The stainings were visualized on 40X magnification in Leica SP8 laser-scanning confocal microscope (Germany).
